# Relaxation Degree Analysis Using Frontal Electroencephalogram Under Virtual Reality Relaxation Scenes

**DOI:** 10.3389/fnins.2021.719869

**Published:** 2021-09-24

**Authors:** Yue Zhang, Lulu Zhang, Haoqiang Hua, Jianxiu Jin, Lingqing Zhu, Lin Shu, Xiangmin Xu, Feng Kuang, Yunhe Liu

**Affiliations:** ^1^School of Electronic and Information Engineering, South China University of Technology, Guangzhou, China; ^2^Department of Psychiatry, Guangzhou First People’s Hospital, The Second Affiliated Hospital, South China University of Technology, Guangzhou, China; ^3^Zhongshan Institute of Modern Industrial Technology of South China University of Technology, Zhongshan, China

**Keywords:** VR, EEG, relaxation state, regression model, machine learning, depression therapy

## Abstract

Increasing social pressure enhances the psychological burden on individuals, and the severity of depression can no longer be ignored. The characteristics of high immersion and interactivity enhance virtual reality (VR) application in psychological therapy. Many studies have verified the effectiveness of VR relaxation therapy, although a few have performed a quantitative study on relaxation state (R-state). To confirm the effectiveness of VR relaxation and quantitatively assess relaxation, this study confirmed the effectiveness of the VR sightseeing relaxation scenes using subjective emotion scale and objective electroencephalogram (EEG) data from college students. Moreover, some EEG features with significant consistent differences after they watched the VR scenes were detected including the energy ratio of the alpha wave, gamma wave, and differential asymmetry. An R-state regression model was then built using the model stacking method for optimization, of which random forest regression, AdaBoost, gradient boosting (GB), and light GB were adopted as the first level, while linear regression and support vector machine were applied at the second level. The leave-one-subject-out method for cross-validation was used to evaluate the results, where the mean accuracy of the framework achieved 81.46%. The significantly changed features and the R-state model with over 80% accuracy have laid a foundation for further research on relaxation interaction systems. Moreover, the VR relaxation therapy was applied to the clinical treatment of patients with depression and achieved preliminary good results, which might provide a possible method for non-drug treatment of patients with depression.

## Introduction

The rapidly developing society enhances pressure on individuals, while mental health problems are getting increasingly critical. By the end of 2020, mental health problems became the second most critical disease worldwide (Randy et al., 2019). Statistics from the World Health Organization in 2020 showed that more than 300 million individuals were suffering from depression, with over 80% not receiving appropriate treatment (WHO, 2020). Patients with mental disorders have a profound negative impact on their personal development, bringing burdens to their families and society (Sidra et al., 2013). Since many studies have confirmed that relaxation could relieve depression (DeBerry, 1982; Lolak et al., 2008), it is highly important to effectively reduce stress and relax.

The current primary methods to relax include deep respiration (Ilse et al., 2014), muscular relaxation (Wesley and Douglas, 1977), music relaxation (Claas et al., 2008; Olga et al., 2011), meditation (Lazar et al., 2000; Narendra et al., 2017), and autogenic training (Ernst and Kanji, 2000). These methods were easily subject to the environment and devices.

The advantages of free space, high immersion, and interactivity have enhanced virtual reality (VR) application in psychological therapy with its rapid development, thus achieving good results (Imran et al., 2014; Allison et al., 2017). Nevertheless, most of these VR scenes are static in nature without scene transitions (Andersen et al., 2017; Kiefl et al., 2018), which might cause boredom and affect the relaxation effect. Moreover, current studies have only made a subjective and qualitative evaluation (Freeman et al., 2017; Linda et al., 2020).

Subjective scales and physiological parameters are generally used to assess the relaxation degree, where the former refers to Perceived Stress Scale (Cohen et al., 1983) and State-trait Anxiety Inventory (Theresa and Hilary, 1992), and the latter includes electroencephalogram (EEG) (Knott et al., 1997; Xing et al., 2019), heart rate variability (Patil and Shirley, 2006; Shu et al., 2020), galvanic skin response (Alexandros et al., 2015), and respiration (Joseph et al., 2016). EEG can relevantly reflect people’s emotional state more accurately among the physiological parameters (Soraia and Manuel, 2019) since emotion is a natural product of neural activity in the brain. Consequently, EEG would be an ideal parameter for measuring relaxation state (R-state), and frontal EEG is considered as the first choice considering its simple operation.

Relevant studies have proved that different frequencies of brain electricity reflect different brain states (Hou et al., 2020), in which alpha, theta, and gamma waves show stronger relevance with R-state. Cahn and Delorme found that the long-term training of Vipassana meditation could increase gamma power (Baruch et al., 2016). Du and Lee observed that low-frequency alpha waves in the left frontal lobe while high-frequency alpha waves in the right frontal lobe increased significantly during positive emotional audio stimulation, where the experimental materials were from the standard International Affective Digital Sounds dataset (Du and Lee, 2015). However, there is a lack of EEG-based relaxation regression models under a VR environment as well as an effective VR relaxation system.

To explore the relaxation effect of VR scenes and the correlation between R-state and frontal EEG, VR relaxation scenes were used as emotion-evoked materials in this study, and a relaxation rating model was established based on EEG data. The VR relaxation scenes and R-state model were then used on patients with depression to explore the possibility of VR relaxation therapy for depression. The study is organized as follows. Section “Materials and Methods” introduces the methods of relaxation VR scene design, data collecting, and model building. Section “Results” shows the results of the analysis of EEG data and the effect of the relaxation model. Section “Application” introduces the application of the research. Section “Discussion” and section “Conclusion” present the discussion and conclusion, respectively.

## Materials and Methods

### Design of Virtual Reality Relaxation Scenes

Four sightseeing-relaxation VR scenes were selected as experimental materials including National Park, Snow Mountain, the Great Wall, and Yunnan. Western classical and new age music were chosen, as background music since O’Sullivan’s research had proved that relaxation music was mainly soft music composed of slow rhythm, low pitch, low volume, beautiful melody, and orchestral instruments (O’Sullivan, 1991), which were copyright free and had been evaluated (Zhu et al., 2019). The selected background music was absolute music without lyrics to avoid cognitive and cultural differences. The description of the scenes and music is shown in Table 1.

**TABLE 1:** Selected VR relaxation scenes.

	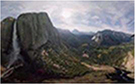	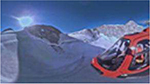	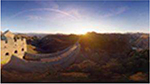	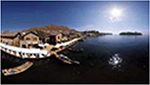
**Sample figure of VR scenes**				
Scene name	National Park	Snow Mountain	The Great Wall	Yunnan
Scene length	199 s	156 s	90 s	144 s
Background music	Pastoral Symphony	Wight Light	Sonata for Spring	The Reiki Gold

*VR, virtual reality.*

**FIGURE 1:**
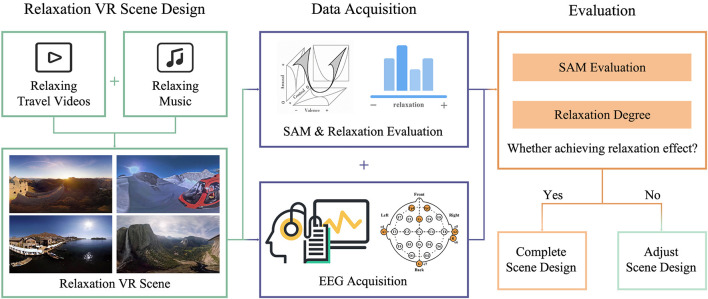
Design process of virtual reality relaxation scenes.

The design process of VR relaxation scenes is illustrated in Figure 1. Appropriate VR scenes and background music were chosen and combined to get visual and auditory fusion materials. EEG was collected during the whole period of watching the VR scene, after which a subjective scale was completed. A designed relaxation VR scene was officially completed when it was verified to achieve an ideal relaxation effect through subjective scale and EEG data evaluation.

### Methods of R-State Evaluation

The forehead prefrontal EEG electrodes of FP1, FP2, and FPZ were chosen to acquire EEG signals for analyzing the relaxation degree of the participants, since the forehead region of the brain was found to be most associated with emotions (Suranjita and Rajesh, 2019).

**FIGURE 2:**
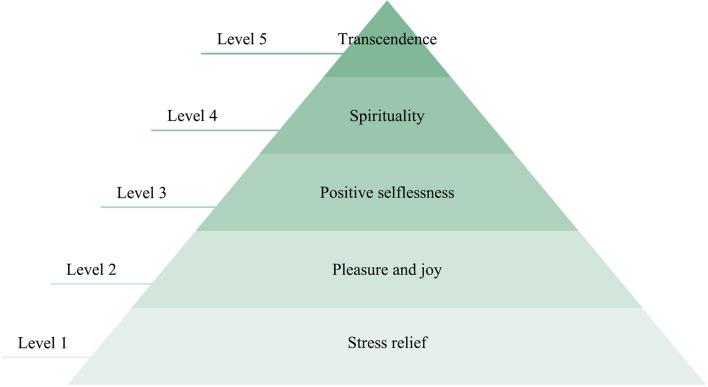
R-state pyramid.

Furthermore, subjective emotion scale Self-Assessment Manikin (SAM) and R-state were used as subjective emotion labels. SAM was based on the valence–arousal–dominance emotion model, which assessed emotion state through three indices. Each score of the three indices ranged from 1 to 9. A higher score indicated a more intense emotion state (higher valence, arousal, and dominance) (Bradley and Lang, 1994; Shu et al., 2018). R-state was based on the R-state pyramid theory proposed by Smith (2005). To keep the grading uniform, the value of the R-state also ranged from 1 to 9. A value of R-state greater than five indicated relaxation, and the numbers 5–9 corresponded to the five levels of R-state, as shown in Figure 2 (value 5 of R-state corresponded to level 1 of stress relief). The higher the score, the more relaxed the participant was.

### Participants and Experimental Procedure

**FIGURE 3:**
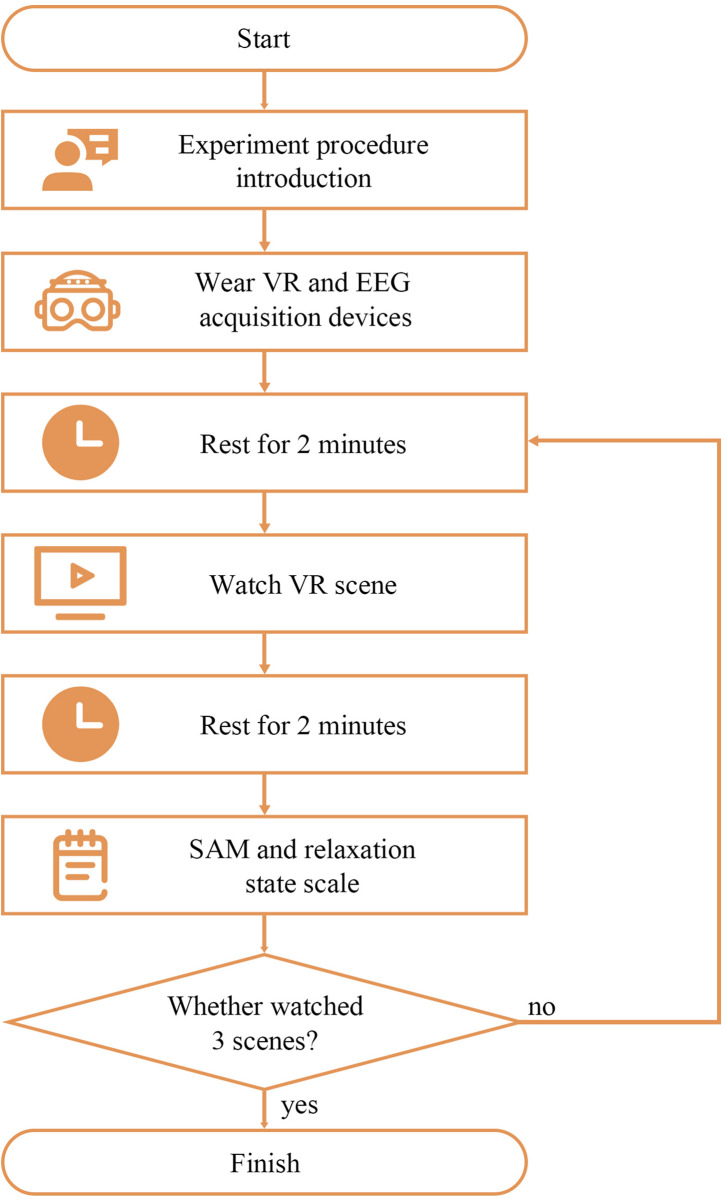
Experimental procedure.

Thirty-three healthy college students (age ranging from 20 to 26 years) including 19 men and 14 women participated in the experiment, with data of only 30 participants valid (16 men and 14 women) for the reason that there were three participants whose EEG data were not fully collected due to the instability of electrode–scalp interface of the EEG collection device. The experiment was conducted in a 30-dB closed soundproof room (Hengqi, Dongguan, China), with experimental equipment, two comfortable chairs, and a table. A pre-training was conducted to make the participants familiar with the experimental steps and SAM scale evaluation method. The procedure of the experiment is shown in Figure 3. After offering personal information and wearing VR glasses together with the EEG acquisition device, participants needed to rest for 2 min with a black screen insight, before and after watching each relaxation scene, the duration of which lasted 90–199 s. Each participant was asked to randomly watch three of the four scenes. The participants were asked to keep their eyes open during the whole experiment to control the variables. The experiment procedure was based on other relevant studies (Zhu et al., 2019). The experimental procedures were approved by the Guangzhou First People’s Hospital (202002030262, on April 1, 2020).

**FIGURE 4:**
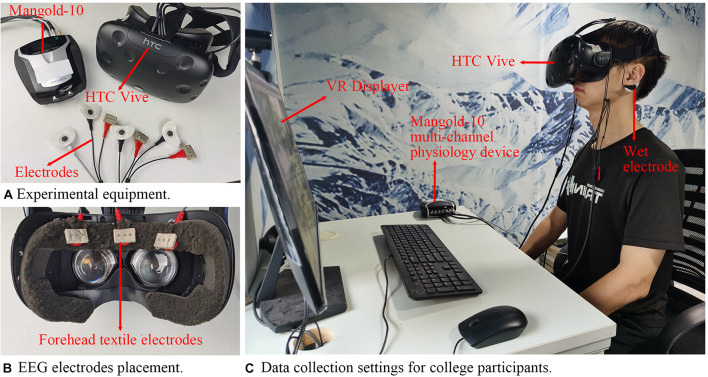
Experimental equipment and settings for college participants.

The VR scenes were watched by HTC Vive, and the EEG acquisition device was a Mangold-10 multichannel physiological instrument with an acquisition frequency of 256 Hz. Figure 4 shows the experimental equipment and the data collection settings for the participants. Three flexible EEG electrodes were embedded in the sponge of the VR device to collect forehead EEG data. The subjective scale was finished after each scene.

### Electroencephalogram Data Processing

**FIGURE 5:**
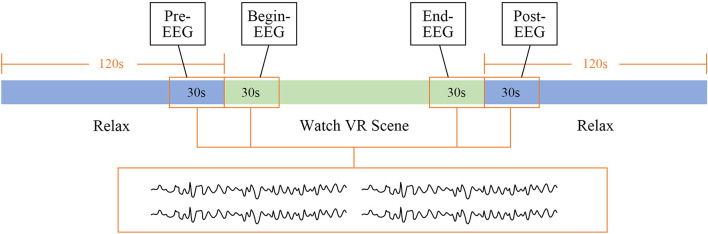
Selected electroencephalogram (EEG) data.

Considering the EEG data collection from watching one section of the VR scene as one segment of data, 80 segments of effective data of normal participants were collected for further analysis (20 segments for each scene). To explore the relaxing effect of the VR scenes, data sections in each segment of EEG data before and after watching scenes for the 30 s (noted as pre EEG and post EEG) and 30-s data during the period of watch VR scenes (noted as begin EEG and end EEG) were selected (Nitin et al., 2016), as shown in Figure 5.

Selected EEG data were filtered using a 1- to 45-Hz Butterworth filter to eliminate the power interference and the baseline drift, after which each segment of de-noised EEG data was decomposed into seven frequency bands according to the different frequencies including delta (δ, 1–3 Hz), theta (θ, 4–7 Hz), low alpha (α_l, 8–10 Hz), high alpha (α_h, 10–12 Hz) (Dombrowe and Hilgetag, 2014), low beta (β_l, 12–20 Hz), high beta (β_h, 20–30 Hz) (Kwon et al., 2011), and gamma waves (γ, 31–50 Hz). Then, EEG features of each band were extracted including the EEG energy value (E), energy ratio (ER), energy entropy (EE), differential entropy (DE), power spectral density, the asymmetry (ASM), rational asymmetry (RASM), differential asymmetry (DASM) of the alpha wave, and the asymmetry of EEG energy ratio of each frequency band, as shown in Table 2 (Jenke et al., 2014; Tortella-Feliu et al., 2014; Zheng and Lu, 2015; Deng et al., 2021).

**TABLE 2:** Selected features.

**Features**	**Selected band**	**Description**
E (energy)	All	Transformed and calculated by Fourier transform
ER (energy ratio)	All	Ratio of EEG energy in different frequency bands ER(ab) = E(a)/E(b)
EE (energy entropy)	All	$EE=\sum_{\text{i}}\log(P_{i}^{2})$
DE (differential entropy)	All	$DE(X)=\frac{1}{2}\log(2\pi e\sigma^{2})$
PSD (power spectral density)	All	$PSD(\text{f})=\frac{\hat{e_{p}}}{\left|1+\sum_{k=1}^{p}\hat{a_{p}}(k)e^{-j2\pi fk}\right|}$
(ASM) Energy asymmetry	Alpha	ASM = E(right) − E(left)
ERASM (energy ratio asymmetry)	All	ERASM = ER(right) − ER(left)
DASM	Alpha	DASM = DE(Xleft) − DE(Xright)
RASM	Alpha	RASM = DE(Xleft)/DE(Xright)

*EEG, electroencephalogram; DASM, differential asymmetry; RASM, rational asymmetry.*

The feature changes of the four data sections extracted from each segment of EEG data including pre–post EEG, pre–end EEG, and begin–end EEG were tested using *t*-test except begin–post EEG for the reason that the mood swings were evident by the VR scene and the goal emotion had not been fully aroused with huge mood swings at the beginning. Moreover, the relaxing emotion would be somewhat diminished during the period of post EEG. Consequently, comparing the begin–post EEG involves multiple variables that cannot be controlled. The features with significant variance (*p* < 0.05) after watching VR scenes were selected for further study. Since participants were exposed to visual and auditory stimuli during the begin EEG period of time, this EEG might be different from pre EEG collected in the resting state. Therefore, both pre–end EEG and begin–end EEG are worth analyzing.

### R-State Model

Since four of the 30 normal participants did not finish the subjective emotion scale, which meant that some of their EEG data lacked an R-state label, and seven segments of the EEG data had obvious noises due to the large body or eye movements during the experiments, only 71 segments of EEG data of 26 participants were used for relaxation model building. After being preprocessed, 147 EEG features were selected to train the R-state model.

First, different lengths of EEG were selected for training. The last 30 and 60 s of EEG data while watching the scenes were chosen to extract selected features for EEG regression model training. Cross-subject research was adopted to make the model more generalized. The leave-one-subject-out (LOSO) method for cross-validation was used to evaluate the accuracy (Tommaso et al., 2006). The LOSO would be performed with n iterations when given a dataset from n participants. The classifier would be trained with EEG data of n − 1 participant and tested on the remaining single subject in each iteration. In this study, the whole segments of EEG data of one participant were considered as one subject data.

#### Data Enhancement

To increase the number of the existing dataset to increase training accuracy, each segment of EEG data was divided into several fragments, and all the fragments in one segment were tagged with the same label. Window sizes that were tried included 2, 4, 6, and 8 s, whereas the overlapping remaining 50%, which meant that the 2-s window corresponded to 1-s step and the 8-s window corresponded to 4-s step. The input data groups are shown in Table 3.

**TABLE 3:** Input data groups.

**Training group**	**EEG length (s)**	**Window size (s)**	**Step length (s)**	**Input data number**
Group 1	30	2	1	2,059
Group 2	30	4	2	994
Group 3	30	6	3	639
Group 4	60	2	1	4,189
Group 5	60	4	2	2,059
Group 6	60	6	3	1,349
Group 7	60	8	4	994

*EEG, electroencephalogram.*

The amount of data per segment of EEG noted as N was calculated by the equation below.


$$N=\frac{L-W}{\text{step}}+1$$

where *L* indicates the length of one segment of the EEG, *W* represents window size, and step is the overlapping length.

#### Regression Model

**FIGURE 6:**
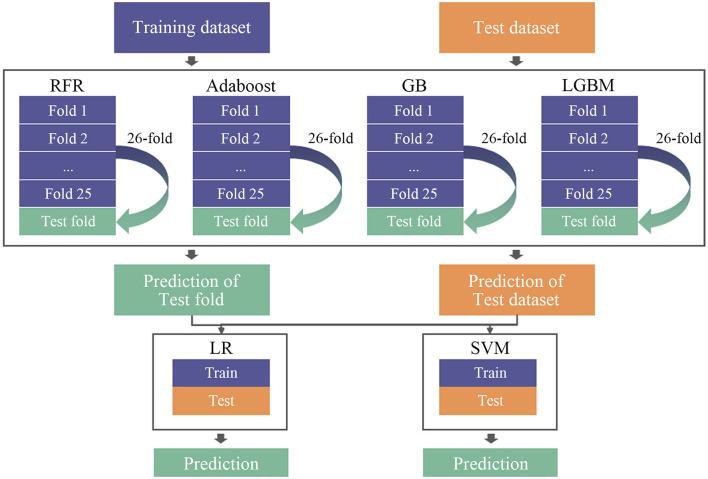
The stacking model diagram.

After all the 147 features from every second of data were extracted and the mean value of each fragment was calculated, the results were then put into different regression models. Eight models were used for a comparison including linear regression (LR), support vector machine (SVM), random forest regression (RFR), adaptive boosting (AdaBoost), BootStrap aggregation (Bagging), gradient boosting (GB), eXtreme GB (XGB), and light GB (LGBM). Stacking regression was then used, which was first proposed by Leo (1996). It was a method that could integrate the outputs of multiple models to produce a new model to improve prediction accuracy. The stacking model generally consists of two levels. Several different high-prediction models with complementary advantages and disadvantages were often used at the first level; and at the second level, one simple model would be used. In this study, four different types of base regression models including RFR, AdaBoost, GB, and LGBM were used at the first level to train the original dataset referring to the results of the eight models training and previous studies (Kim et al., 2020). RFR and boosting models are the most commonly used models at the first level of stacking because these two models belong to the parallel model and the serial model, which are quite different and have generalization to the results. And at the second level, a simple model such as LR or SVM will be used to integrate the results of the models used at the first level to prevent overfitting (David, 1992; Bohdan, 2020). LOSO method was also used in the first level so that 26-fold cross-validation would be done by each model to get predicted labels. The predictions of each test fold were then put into the second level as the training dataset, and the average of the 26-fold predictions would be taken as test datasets in the second level. Two simple models LR and SVM were tried at the second level to make a comparison. The diagram of model stacking in the regression work is shown in Figure 6.

#### Evaluation Index

Mean absolute error (MAE) and mean relative accuracy (ACC) (Li et al., 2019) were used as indices to evaluate the results of different model training. MAE calculated the absolute error between the predicted value and the true value. The formula is illustrated below, where n indicates the number of the EEG data, *y*_*i*_ is the true value, and $\hat{y_{i}}$ is the predicted value. The lower the value, the better is the training model.


$$M\,A\,E=\frac{1}{\text{n}}\,\sum_{k=0}^{n}\left|\left(y_{i}-\hat{y_{i}}\right)\right|$$

The calculation formula of ACC is shown below with an index ranging in value from 0 to 1. The closer the value is to 1, the better is the training model. ACC reflected the relative error between the predicted and true values, which would be more comparable than MAE.


$$A\,C\,C=1-\frac{1}{n}\,\sum_{k=0}^{n}\frac{\left|\left(y_{i}-\hat{y_{i}}\right)\right|}{y_{i}}$$

## Results

### Subjective Emotion Scale Result

**FIGURE 7:**
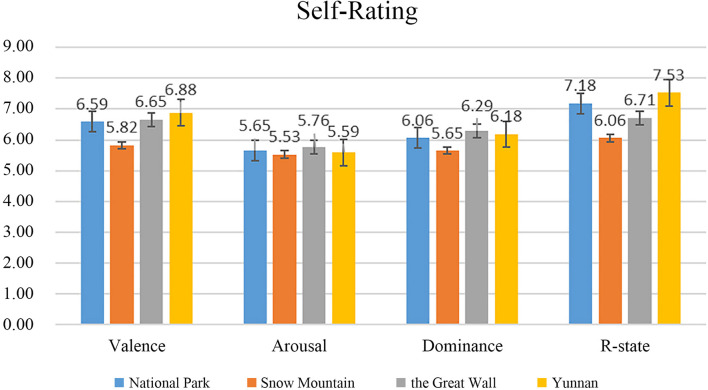
Self-rating scale of normal participants.

The result of the subjective scale is shown in Figure 7. Since the relaxation degree score greater than five indicated that the scene had a relaxing effect, all the four relaxation scenes were effective (National Park 7.18, Snow Mountain 6.06, the Great wall 6.71, and Yunnan 7.53), in which Yunnan was the most relaxing VR scene. Furthermore, the results also showed that with increase in the relaxation degree, the value of valence also increased.

### Electroencephalogram Feature Analysis Result

The typically changed EEG features of the participants are shown in Table 4 including energy features, energy ratio features, and SE features of each band.

**TABLE 4:** EEG features with significant variance.

**Begin–end**		**Pre–post**		**Pre–end**			
**Feature**	**p**	**Feature**	**p**	**Feature**	**p**	**Feature**	**p**
dsp_alpha_l2	0.03240	theta1/alpha_l1	0.00450	SE_theta3	0.00008	EE_theta1	0.01359
gamma3	0.03790	EE_delta2	0.01011	EE_theta2	0.00013	alpha1_h/gamma1	0.01521
gamma2	0.03956	delta1/alpha_l1	0.01133	EE_theta3	0.00016	alpha3_h/gamma3	0.02441
alpha_h3	0.03968	delta1/theta1	0.01970	SE_theta2	0.00021	delta2/theta2	0.02842
dsp_beta_h3	0.04018	EE_alpha_l1	0.02283	SE_alpha_h2	0.00079	alpha2_h/gamma2	0.03056
delta3	0.04132	delta2/alpha_l2	0.02962	SE_alpha_h1	0.00080	alpha_h1/beta_h1	0.03092
beta_l3	0.04230	EE_beta_h3	0.03087	SE_alpha_h3	0.00083	EE_alpha_h1	0.03184
		SE_beta_l1	0.03722	alpha_h3/beta_l3	0.00152	alpha_h3/beta_h3	0.04503
		SE_beta_h3	0.03838	alpha_h1/beta_l1	0.00170	delta1/beta_l1	0.04796
		EE_delta3	0.03839	alpha_h2/beta_l2	0.00286	EE_alpha_h3	0.04892
		EE_beta_h2	0.03896	SE_delta3	0.00916		
		SE_delta2	0.04095	SE_theta1	0.00949		

*EEG, electroencephalogram.*

**FIGURE 8:**
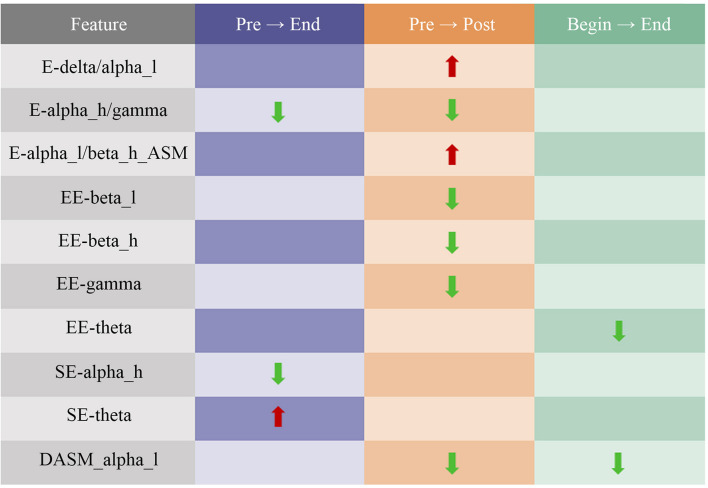
Summary of changes in electroencephalogram (EEG) characteristics of normal participants after watching the relaxation scenes.

The results of pre EEG to end EEG, pre EEG to post EEG, and begin EEG to end EEG were used as comparison groups. Ten features were found from significantly changed features, as was shown in Figure 8, to have consistency differences after watching the VR relaxation scenes, which meant the EEG feature values of all the 26 participants showed an increasing or a decreasing trend after watching the VR scene for each participant. The significantly changed features included E-delta/alpha_l, E-alpha_h/gamma, E-alpha_l/beta_h-ASM, EE-beta_l, EE-beta_h, EE-gamma, EE-theta, SE-alpha_h, SE-theta, and DASM-alpha_l. Moreover, features E-delta/alpha_l, E-alpha_l/beta_h-ASM, and SE-theta showed an increasing trend after watching the relaxation VR scene. Features E-alpha_h/gamma, EE-beta_l, EE-beta_h, EE-gamma, SE-alpha_h, SE-theta, and DASM-alpha_l showed a decreasing trend.

### Relaxation Regression Results

Table 5 shows the ACC results of the different training models in each group. From the average accuracy results of the different training models in each group, it could be observed that the accuracy of XGB and LGBM reached above 80%, and LGBM got the best result of 80.42% on average. While using LGBM to train the model, Group 1 performed the best, with the accuracy of 80.69%.

**TABLE 5:** Relaxation model training ACC results.

	**Group 1**	**Group 2**	**Group 3**	**Group 4**	**Group 5**	**Group 6**	**Group 7**	**AVE**
LR	0.79934	0.80281	0.79370	0.70145	0.63516	0.41623	0.34172	0.64149
SVM	0.78001	0.78299	0.78501	0.77675	0.78107	0.78072	0.781061	0.78102
RF	0.80029	0.79645	0.80021	0.80219	0.80079	0.79871	0.79849	0.79959
AdaBoost	0.78393	0.78701	0.78949	0.78143	0.78367	0.78859	0.79119	0.78647
Bagging	0.80111	0.80163	0.79669	0.80190	0.79709	0.80195	0.79637	0.79953
GB	0.79695	0.79989	0.80222	0.79602	0.79792	0.79721	0.79539	0.79794
XGB	0.80199	0.80354	0.80199	0.80317	0.80138	0.79588	0.79755	0.80079
LGBM	**0.80692**	0.80431	0.80253	0.80519	0.80538	0.80277	0.80237	**0.80421**

*ACC, mean relative accuracy; LR, linear regression; SVM, support vector machine; RF, random forest; GB, gradient boosting; XGB, eXtreme gradient boosting; LGBM, light gradient boosting. The bold values indicate the best experimental results.*

Mean absolute error result is shown in Table 6. It could be found that in using LGBM to train Group 1, the lowest value of 1.00494 was obtained. While comparing the results of each group, it could be found that in general, using 30 s of EEG data (Groups 1, 2, and 3) to train the model would get better results than 60 s of EEG data (Groups 4, 5, 6, and 7). Furthermore, the model stacking method was applied to train Group 1. The result is shown in Table 7. It could be found that using stacking increased the accuracy of the predictions by approximately 1% and decreased the MAE values by 1, which indicated that the model was optimized. Moreover, using SVM at the second level got better results than LR.

**TABLE 6:** Relaxation model training MAE results.

	**Group 1**	**Group 2**	**Group 3**	**Group 4**	**Group 5**	**Group 6**	**Group 7**	**AVE**
LR	1.02831	1.02005	1.09353	1.65507	2.10446	3.56199	4.04282	2.07232
SVM	1.07132	1.06300	1.05260	1.08742	1.06601	1.06833	1.06866	1.06819
RF	1.03068	1.05419	1.04110	1.02747	1.03011	1.03923	1.05331	1.03944
AdaBoost	1.18253	1.12698	1.09482	1.20856	1.18663	1.12918	1.10883	1.14822
Bagging	1.02489	1.03342	1.06349	1.03192	1.04542	1.04085	1.07164	1.04452
GB	1.04648	1.03242	1.03109	1.05666	1.05017	1.05481	1.06062	1.04746
XGB	1.05373	1.06005	1.07795	1.04851	1.06283	1.09146	1.09887	1.07049
LGBM	**1.00494**	1.01620	1.03201	1.01564	1.01933	1.02982	1.03269	**1.02152**

*MAE, mean absolute error; LR, linear regression; SVM, support vector machine; RF, random forest; GB, gradient boosting; XGB, eXtreme gradient boosting; LGBM, light gradient boosting. The bold values indicate the best experimental results.*

**TABLE 7:** Stacking model results.

**Second level model**	**MAE**	**ACC**
LR	0.98942	0.81216
SVM	**0.46846**	**0.81462**

*MAE, mean absolute error; ACC, mean relative accuracy; LR, linear regression; SVM, support vector machine. The bold values indicate the best experimental results.*

## Application

**FIGURE 9:**
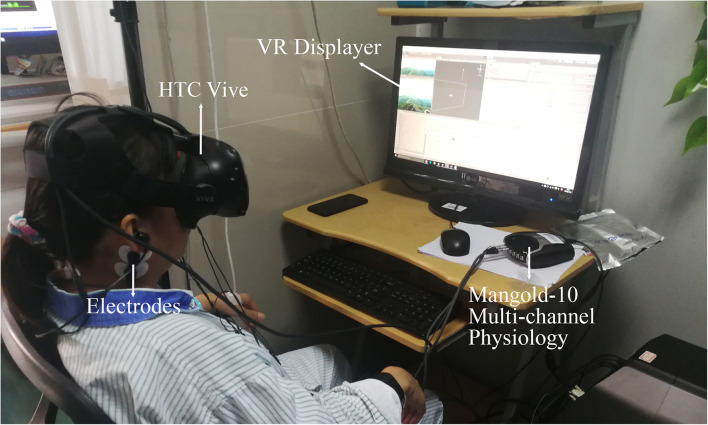
Data collection settings for patients with depression.

The VR relaxation scenes were applied to assist in the treatment of patients with depression in the Guangzhou First People’s Hospital. Twenty-two patients with first-episode depression including six men and 16 women (age ranging from 19 to 50 years) volunteered for the VR treatment. Each patient was asked to watch only one VR scene, the Great Wall, to avoid discomfort caused by watching VR for a long time. EEG was also acquired during the procedure, and patients were asked to verbally answer how they felt after watching. Figure 9 shows the data collection settings for the patients, who were asked to sit and watch the VR scene wearing the VR glasses in front of a table on which there was a computer monitor and the EEG acquisition device. Written consent was obtained from each participant before the experiment.

Patients’ subjective answer results are shown in Table 8. It could be seen that most patients with depression felt relaxed after watching the VR scene except for two patients.

**TABLE 8:** Relaxation model training MAE results.

**Relaxation state**	**Subject serial number**	**Total number**	**Male number**	**Female number**
Very relaxed	Subject 1, 3, 4, 5–10, 15, 20, 22	12	1	11
A little relaxed	Subject 2, 11, 13, 14, 17, 18, 19, 21	8	4	4
No relaxed	Subject 12, 16	2	1	1

*MAE, mean absolute error.*

The EEG datasets of the depression patients were preconditioned in the same manner as those of normal college students. After preprocessing, the last 30-s EEG data while watching the relaxation scene were put into the stacking regression model whose second layer was SVM to predict the R-state to demonstrate the effectiveness of the VR relaxation scene to depression patients. The method of EEG data progress was the same as that of Group 1, of which the R-state prediction result was the best. The predicted R-state results of disorder patients are shown in Table 9, demonstrating that all the prediction values were over 5, and the average of the predicted R-state was 6.54, which was close to the subjective rating value (6.71). These results confirmed that the VR relaxation scene has a positive effect on the relaxation therapy of patients with depression.

**TABLE 9:** Predicted R-state of each patient.

**Subject number**	**Predicted R-state**	**Subject number**	**Predicted R-State**
Subject 1	7.15	Subject 12	5.76
Subject 2	6.32	Subject 13	7.13
Subject 3	6.55	Subject 14	6.64
Subject 4	7.36	Subject 15	6.63
Subject 5	7.11	Subject 16	5.66
Subject 6	5.92	Subject 17	5.68
Subject 7	7.09	Subject 18	6.18
Subject 8	6.64	Subject 19	6.53
Subject 9	6.82	Subject 20	5.51
Subject 10	7.01	Subject 21	6.56
Subject 11	6.68	Subject 22	6.85
Average result		6.54	

**FIGURE 10:**
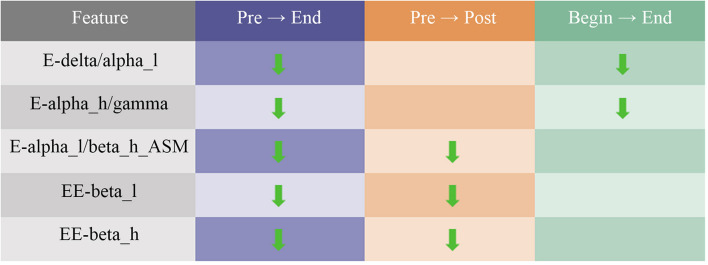
Summary of changes in electroencephalogram (EEG) characteristics of patients with depression after the relaxation scenes.

The EEG features of patients with depression were analyzed in the same manner as those of normal participants. Five features had significant consistency differences after watching the VR relaxation scenes, including E-delta/beta_l, E-delta/beta_h, E-alpha_l/gamma, E-alpha_h/gamma, and E-beta_l/gamma, as shown in Figure 10. All of these changing features were on a downward trend. Moreover, E-alpha_h/gamma had the same trend as that of normal participants.

## Discussion

The subjective evaluations in the normal participants and patients with depression demonstrated that the sightseeing-relaxation VR scenes with new age music had relaxing effects. However, it was hard to say whether the visual scene or the auditory music had a greater effect on relaxation. Some studies have found that auditory stimulations aroused emotions much better than visual materials. Therefore, the impact of relaxation VR scenes and relaxing background music must be explored.

Different data processing methods were used to analyze the EEG datasets for R-state study in this study. The length of chosen EEG was 30 s including sections during watching VR scenes and during the 2-min relaxing period. In the previous study, 60 s of EEG data of one section before and after watching the VR scenes were used (Zhu et al., 2019). However, since the participant’s emotion was easily influenced by other psychological activities during 2 min of relaxation before and after watching the VR scene, the 60 s of EEG data was relatively long. While training the R-state model, using 30 s of EEG data also showed higher accuracy and lower MAE than that at 60 s, consistent with Kumar’s research (Nitin et al., 2016).

From the EEG feature analysis results, it could be found that most of the distinctive features were theta, alpha, beta, and gamma waves, which were consistent with Cahn’s research (Baruch et al., 2016). The appearance of the beta wave was associated with mental tension and emotional excitement. When people felt relaxed, the energy and entropy of the beta wave should go down. As a result, the feature values of EE-beta_h and EE-beta_l went down. Moreover, since many studies have found that meditation and relaxation could increase gamma wave, the value of E-alpha_l/gamma and E-alpha_h/gamma increased after watching relaxation scenes. The significant variance in gamma-related features also indicated that the relaxation effects of the sightseeing scenes used in the experiment might be similar to those of meditation.

Moreover, it could be easily detected from Figure 10 that all the features with significant differences among the group of patients with depression were energy ratios, most of which were beta- and gamma-related features. It was probably because depression varied widely among individuals, and the ratio-related features could neutralize some of the individual differences (Kan and Lee, 2015). Moreover, since patients with depression felt stressed more easily, beta wave, which was associated more with anxiety, would more likely to be affected by relaxation scenes. As per Smith’s theory, reducing stress enhanced relaxation (Smith, 1988). Many studies have proved that patients with depression had increased alpha (Hosseinifard et al., 2013) and beta power (Clark et al., 2016). The decreased features of E-alpha_l/gamma, E-alpha_h/gamma, and E-beta_l/gamma might also have confirmed the effectiveness of VR-relaxation therapy in treating depression. When comparing the changes of EEG features between normal people and patients with depression, it could be found that there existed one feature, E-alpha_h/gamma, having the same trend as that of normal participants. Related studies have found that the gamma wave and alpha wave of normal people and patients with depression are relatively sensitive (Grey et al., 2010), which might cause the same significant changes. Although the findings have not been medically proven yet, these results have provided a reference for future studies.

It could be found from Table 9 that all the EEG data of depression patients predicted a level of R-state greater than 5, which meant that the emotions of all the patients were predicted to be relaxed. However, two patients were not relaxed in their subjective assessment. This difference might be due to the fact that the relaxation model was based on EEG data from normal people. Since the R-state model was built by datasets of normal people, and there existed some differences between the EEG of normal individuals and patients with depression (Davidson et al., 2002; Acharya et al., 2015), using the R-state model to predict people with depression might not be particularly accurate. However, one feature with the same trend when watching the relaxation scenes in these two groups was found, and the predicted results of the R-state still have reference value. Therefore, it is necessary to train the relaxation model using the EEG data and relaxation label of patients with depression. In further research, more experiments would be conducted covering college students, patients with depression, and some other groups of people, to verify the application scopes of the relaxation model.

## Conclusion

In this study, VR relaxation scenes were used to promote the R-state for college students. Some EEG features were found to have a consistent significant trend of variance among different participants while watching the relaxation scenes, including EE-gamma, E-alpha_h/gamma, and DASM. These significantly changed features provide a reference for optimizing the relaxation prediction model and relaxation interaction system research based on EEG in the future. Eight machine learning models including LR, SVM, and LGBM were conducted to train the R-state regression model, and the LOSO method for cross-validation was used to evaluate the results. The mean accuracy reached approximately 80.42% using the LGBM model. Model stacking methods were then applied to optimize the model. The mean accuracy of the framework achieved approximately 81.46%, which increased by approximately 1%. The VR relaxation scenes were then used to help with the treatment of patients with depression, which have received good results. This work provides an objective index reference for the evaluation and treatment of depression using VR relaxation scenes and also explores the feasibility of VR relaxation scenes in the adjuvant treatment of depression.

## Data Availability Statement

The raw data supporting the conclusions of this article will be made available by the authors, without undue reservation.

## Ethics Statement

The studies involving human participants were reviewed and approved by the Guangzhou First People’s Hospital (202002030262, on April 1 2020). The patients/participants provided their written informed consent to participate in this study.

## Author Contributions

YZ and LLZ were responsible for the entire study, including the study concept, the study design, and the application. YZ, HH, JJ, and LQZ contributed to the VR scene design. YZ, JJ, LS, and XX were responsible for the EEG collection design and data analysis. FK and YL helped with the EEG collection. All authors listed have made a direct and intellectual contribution to the work.

## Conflict of Interest

The authors declare that the research was conducted in the absence of any commercial or financial relationships that could be construed as a potential conflict of interest.

## Publisher’s Note

All claims expressed in this article are solely those of the authors and do not necessarily represent those of their affiliated organizations, or those of the publisher, the editors and the reviewers. Any product that may be evaluated in this article, or claim that may be made by its manufacturer, is not guaranteed or endorsed by the publisher.
