# Supplementary material for: Relaxation Degree Analysis Using Frontal Electroencephalogram Under Virtual Reality Relaxation Scenes
Source: Front Neurosci. 2021 Sep 24;15:719869. doi: 10.3389/fnins.2021.719869 (PMC8500181; doi:10.3389/fnins.2021.719869)
Supplement: Supplementary file 1 [file Data_Sheet_1.docx]

Supplementary Material

# Supplementary Tables

TABLE I

Selected VR Relaxation Scenes

| Sample figure of VR scenes | 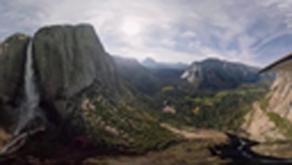 | 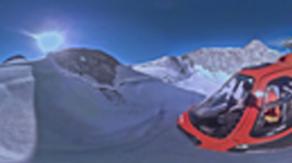 | 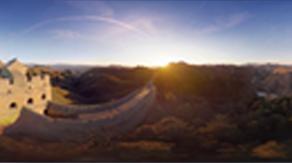 | 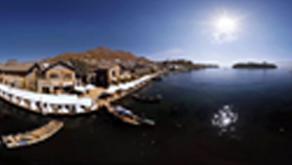 |  |
| --- | --- | --- | --- | --- | --- |
| Scene name | National Park | Snow Mountain | The Great Wall | Yunnan |  |
| Scene length | 199s | 156s | 90s | 144s |  |
| Background music | Pastoral Symphony | Wight Light | Sonata for Spring | The Reiki Gold |  |

TABLE II

Selected Features

| Features | Selected Band | Description |
| --- | --- | --- |
| E(Energy) | All | Transformed and calculated by Fourier transform |
| ER(Energy Ratio) | All | Ratio of EEG energy in different frequency bands  ER(ab) = E(a)/E(b) |
| EE (Energy Entropy) | All | $EE=\sum_{\text{i}} \log(P_{i}^{2})$ |
| DE(Differential Entropy) | All | $DE(X)=\frac{1}{2}\log(2\pi e\sigma^{2})$ |
| PSD(Power Spectrum Density) | All | $PSD(\text{f})=\frac{\widehat{e_{p}}}{\vert1+\sum_{k=1}^{p} \widehat{a_{p}}(k)e^{-j2\pi fk}\vert}$ |
| (ASM)Energy Asymmetry | Alpha | ASM = E(right)-E(left) |
| ERASM (Energy Ratio Asymmetry) | All | ERASM = ER(right)-ER(left) |
| DASM | Alpha | DASM = DE(Xleft)-DE(Xright) |
| RASM | Alpha | RASM = DE(Xleft)/DE(Xright) |

TABLE IV

EEG Features with Significant Variance

| begin-end | | pre-post | | Pre-end | | | |
| --- | --- | --- | --- | --- | --- | --- | --- |
| Feature | P | Feature | P | Feature | P | Feature | P |
| dsp_alpha_l2 | 0.03240 | theta1/alpha_l1 | 0.00450 | SE_theta3 | 0.00008 | EE_theta1 | 0.01359 |
| gamma3 | 0.03790 | EE_delta2 | 0.01011 | EE_theta2 | 0.00013 | alpha1_h/gamma1 | 0.01521 |
| gamma2 | 0.03956 | delta1/alpha_l1 | 0.01133 | EE_theta3 | 0.00016 | alpha3_h/gamma3 | 0.02441 |
| alpha_h3 | 0.03968 | delta1/theta1 | 0.01970 | SE_theta2 | 0.00021 | delta2/theta2 | 0.02842 |
| dsp_beta_h3 | 0.04018 | EE_alpha_l1 | 0.02283 | SE_alpha_h2 | 0.00079 | alpha2_h/gamma2 | 0.03056 |
| delta3 | 0.04132 | delta2/alpha_l2 | 0.02962 | SE_alpha_h1 | 0.00080 | alpha_h1/beta_h1 | 0.03092 |
| beta_l3 | 0.04230 | EE_beta_h3 | 0.03087 | SE_alpha_h3 | 0.00083 | EE_alpha_h1 | 0.03184 |
|  |  | SE_beta_l1 | 0.03722 | alpha_h3/beta_l3 | 0.00152 | alpha_h3/beta_h3 | 0.04503 |
|  |  | SE_beta_h3 | 0.03838 | alpha_h1/beta_l1 | 0.00170 | delta1/beta_l1 | 0.04796 |
|  |  | EE_delta3 | 0.03839 | alpha_h2/beta_l2 | 0.00286 | EE_alpha_h3 | 0.04892 |
|  |  | EE_beta_h2 | 0.03896 | SE_delta3 | 0.00916 |  |  |
|  |  | SE_delta2 | 0.04095 | SE_theta1 | 0.00949 |  |  |

TABLE III

Input Data Groups

| Training group | EEG length(s) | Window size(s) | Step length(s) | Input data number |
| --- | --- | --- | --- | --- |
| Group 1 | 30 | 2 | 1 | 2059 |
| Group 2 | 30 | 4 | 2 | 994 |
| Group 3 | 30 | 6 | 3 | 639 |
| Group 4 | 60 | 2 | 1 | 4189 |
| Group 5 | 60 | 4 | 2 | 2059 |
| Group 6 | 60 | 6 | 3 | 1349 |
| Group 7 | 60 | 8 | 4 | 994 |

TABLE VI

Relaxation Model Training MAE Results

|  | Group 1 | Group 2 | Group 3 | Group 4 | Group 5 | Group 6 | Group 7 | AVE |
| --- | --- | --- | --- | --- | --- | --- | --- | --- |
| LR | 1.02831 | 1.02005 | 1.09353 | 1.65507 | 2.10446 | 3.56199 | 4.04282 | 2.07232 |
| SVM | 1.07132 | 1.06300 | 1.05260 | 1.08742 | 1.06601 | 1.06833 | 1.06866 | 1.06819 |
| RF | 1.03068 | 1.05419 | 1.04110 | 1.02747 | 1.03011 | 1.03923 | 1.05331 | 1.03944 |
| AdaBoost | 1.18253 | 1.12698 | 1.09482 | 1.20856 | 1.18663 | 1.12918 | 1.10883 | 1.14822 |
| Bagging | 1.02489 | 1.03342 | 1.06349 | 1.03192 | 1.04542 | 1.04085 | 1.07164 | 1.04452 |
| GB | 1.04648 | 1.03242 | 1.03109 | 1.05666 | 1.05017 | 1.05481 | 1.06062 | 1.04746 |
| XGB | 1.05373 | 1.06005 | 1.07795 | 1.04851 | 1.06283 | 1.09146 | 1.09887 | 1.07049 |
| LGBM | **1.00494** | 1.01620 | 1.03201 | 1.01564 | 1.01933 | 1.02982 | 1.03269 | **1.02152** |

TABLE V

Relaxation Model Training ACC Results

|  | Group 1 | Group 2 | Group 3 | Group 4 | Group 5 | Group 6 | Group 7 | AVE |
| --- | --- | --- | --- | --- | --- | --- | --- | --- |
| LR | 0.79934 | 0.80281 | 0.79370 | 0.70145 | 0.63516 | 0.41623 | 0.34172 | 0.64149 |
| SVM | 0.78001 | 0.78299 | 0.78501 | 0.77675 | 0.78107 | 0.78072 | 0.781061 | 0.78102 |
| RF | 0.80029 | 0.79645 | 0.80021 | 0.80219 | 0.80079 | 0.79871 | 0.79849 | 0.79959 |
| AdaBoost | 0.78393 | 0.78701 | 0.78949 | 0.78143 | 0.78367 | 0.78859 | 0.79119 | 0.78647 |
| Bagging | 0.80111 | 0.80163 | 0.79669 | 0.80190 | 0.79709 | 0.80195 | 0.79637 | 0.79953 |
| GB | 0.79695 | 0.79989 | 0.80222 | 0.79602 | 0.79792 | 0.79721 | 0.79539 | 0.79794 |
| XGB | 0.80199 | 0.80354 | 0.80199 | 0.80317 | 0.80138 | 0.79588 | 0.79755 | 0.80079 |
| LGBM | **0.80692** | 0.80431 | 0.80253 | 0.80519 | 0.80538 | 0.80277 | 0.80237 | **0.80421** |

TABLE VIII

Relaxation Model Training MAE Results

| Relaxation state | Subject serial number | Total number | Male number | Female number |
| --- | --- | --- | --- | --- |
| Very relaxe | Subject 1,3,4,5-10,15,20,22 | 12 | 1 | 11 |
| A little relaxed | Subject 2,11,13,14,17,18,19,21 | 8 | 4 | 4 |
| No relaxed | Subject 12,16 | 2 | 1 | 1 |

TABLE VII

Stacking Model Results

| Second Level Model | MAE | ACC |
| --- | --- | --- |
| LR | 0.98942 | 0.81216 |
| SVM | 0.46846 | 0.81462 |

TABLE IX

Predicted R-stare of Each Patient

| Subject  Number | Predicted  R-State | Subject  Number | Predicted  R-State |
| --- | --- | --- | --- |
| Subject 1 | 7.15 | Subject 12 | 5.76 |
| Subject 2 | 6.32 | Subject 13 | 7.13 |
| Subject 3 | 6.55 | Subject 14 | 6.64 |
| Subject 4 | 7.36 | Subject 15 | 6.63 |
| Subject 5 | 7.11 | Subject 16 | 5.66 |
| Subject 6 | 5.92 | Subject 17 | 5.68 |
| Subject 7 | 7.09 | Subject 18 | 6.18 |
| Subject 8 | 6.64 | Subject 19 | 6.53 |
| Subject 9 | 6.82 | Subject 20 | 5.51 |
| Subject 10 | 7.01 | Subject 21 | 6.56 |
| Subject 11 | 6.68 | Subject 22 | 6.85 |
| Average Result | | 6.54 | |
